# Emphasis on Early Identification of Risk Factors to Curtail High Mortality Involved With Ischemic Colitis (IC) After Abdominal Aortic Aneurysm (AAA) Repair

**DOI:** 10.7759/cureus.23492

**Published:** 2022-03-25

**Authors:** Shashwat Shrivastava, Shitij Shrivastava, Supraja Naidu Avula, Anusheel ., Vishwanath Thondamala, Chibuzor V Onuchukwu, Lubna Mohammed

**Affiliations:** 1 Vascular Surgery, California Institute of Behavioral Neurosciences & Psychology, Fairfield, USA; 2 Interventional Cardiology, California Institute of Behavioral Neurosciences & Psychology, Fairfield, USA; 3 Orthopedics and Traumatology, California Institute of Behavioral Neurosciences & Psychology, Fairfield, USA; 4 General Practice, California Institute of Behavioral Neurosciences & Psychology, Fairfield, USA; 5 Pulmonary and Critical Care Medicine, California Institute of Behavioral Neurosciences & Psychology, Fairfield, USA; 6 Public Health, California Institute of Behavioral Neurosciences & Psychology, Fairfield, USA; 7 Internal Medicine, California Institute of Behavioral Neurosciences & Psychology, Fairfield, USA

**Keywords:** abdominal aortic aneurysm, abdominal aortic aneurysm repair, intact aneurysm, ruptured aneurysm, endovascular repair, open repair, gastrointestinal complications, colonic ischemia, ischemic colitis

## Abstract

Ischemic colitis (IC) is one of the most feared complications after abdominal aortic aneurysm (AAA) repair. Though the complication is seen in only a handful of total repairs, the mortality rates after IC is very high. Due to infrequent presentation, attending doctors may overlook this possibility leading to a delay in diagnosis. Open repair and endovascular aneurysm repair (EVAR) are the two most common methods used for repairing aneurysms and both of these procedures have their implications in the development of IC. While the incidence of IC was greater after open repair, it also harbored more patients with ruptured aneurysms, emergency repairs, and patients in shock. Similarly, a significant proportion of patients having ruptured aneurysms developed IC. Controlling minor variables like acidosis, hypothermia, hypovolemia, and clamp time duration can play a cumulative role in diminishing this hazardous complication. Medical advancements and minimally invasive technologies have improved the quality of care and operation success considerably, but researchers have not identified a statistically significant association in the prevention of postoperative IC. Therefore, early diagnosis and prompt management become crucial in reducing mortality rates. This can be achieved by being aware of impending signs and symptoms especially in patients with risk factors and being proactive in medical management.

## Introduction and background

Abdominal aortic aneurysm (AAA) is seen very commonly in elderly males and Caucasian ethnicity. 30% of asymptomatic AAA are generally diagnosed as a pulsatile mass on physical examination. This condition arises due to global dilation and degeneration of the connective tissue of the aortic wall because of the combined effect of several risk factors, most commonly smoking, hypertension, and atherosclerosis. Genetic factors, age-related proteolytic degradation, chronic stress on the wall, all contribute to the development of AAA [1]. To avoid the risk of rupture, several guidelines have been implemented by the United States Preventive Services Task Force (USPSTF). The USPSTF recommends a one-time screening for AAA with ultrasonography in elderly men with an age range from 65 to 75 who have smoked even once in their lifetime [2].

Ischemic Colitis (IC) is a disease prevalent in patients undergoing infrarenal AAA repair. The anatomical orientation of this aneurysm increases the possibility of developing IC. Cross-clamping of the aorta, prolonged hypotension, microembolization, colonic trauma during open repair, and reperfusion injury are some of the possible etiologies for the presenting complication. Reduction in perfusion of the inferior mesenteric artery (IMA) can produce ischemia, most commonly on the splenic flexure and rectosigmoid junction due to frail anastomoses between the blood vessels [3]. Consequently, it can produce life-threatening sequela and contribute to prolonged length of intensive care unit (ICU) stays and accentuate healthcare costs. According to a study, 49.3% of elderly patients having prolonged bowel ischemia required surgical resection (laparotomy) of the involved colonic segment, worsening the clinical outcomes, whereas 50.7% were managed medically [4].

AAA is repaired through open or endovascular aneurysm repair (EVAR). IC has always been dreaded as one of the most life-threatening complications, post infrarenal AAA repairs with mortality reaching a stark number of 38.7% [3,4]. Very little research is available on the efficacy of new advancements in vascular repairs to reduce the chance of postoperative IC, due to the small sample size. Furthermore, the effect of IC on short-term and long-term mortality is not very well understood [5]. Due to the unavailability of established studies on the latest interventional technology, the likelihood of treatment success is vastly relied on being mindful of risk factors and recognizing early signs and symptoms.

Since IC encompasses only 2.2% of the total post-repair complications, the awareness among the clinicians is modest [4]. The purpose of this literature review is to identify the risk factors and underlying processes behind the development of IC in patients following open or endovascular AAA repair. Reemphasizing necessary attention towards this catastrophic complication can facilitate early diagnosis and intervention which is essential for a substantial reduction in the death rates. Identification of important risk factors in patients scheduled for AAA repair can allow a prospective watch for this devastating disease.

## Review

An observational study, with a sample size of 446 patients conducted by Dr. Latz in the year 2020, evaluated the prevalence of mortality between open and endovascular approaches for aortic aneurysm repair. This study was prioritized for review because of the large sample size, which is generally a concern when studying complications of AAA and its recency. The risk of mortality was studied within 30 days of the intervention. The anatomical orientation of the aneurysm served as the basis for choosing the most suitable approach. Aneurysms originating close to the infrarenal artery (juxtarenal aneurysms) were operated through open repair whereas pararenal aneurysms were treated endovascularly. Mortality within 30 days was seen in 135 patients which contributed to 30.5% of the total repairs. 25 deaths were reported with EVAR, whereas the deaths in open repair were 110. There was a statistically significant association between open repair in complicated abdominal aortic aneurysms (cAAAs) and the development of IC, leading to longer ICU stays [6]. A meta-analysis and meta-regression analysis performed by Dr. Nikolaos also asserts the increased popularity of deaths in open repairs in contrast to endovascular repair. EVAR was associated with reduced perioperative mortality, especially for ruptured aneurysms (odds ratio (OR) 0.54, 95% confidence interval (CI) 0.51-0.57, p < .001). The data displayed a significant association, but the randomized trials failed to demonstrate causality between EVAR and reduced perioperative mortality [7]. The Society for Vascular Surgery recommends EVAR as the primary choice for AAA repair, despite acknowledging the scarcity of strong evidence [7].

The important thing to be noted is that the differences in mortality between two repairs are relevant when deaths within 30 days are considered. A meta-analysis of long-term outcomes of open and endovascular repairs established no difference in all-cause mortality rates between the two groups. The long-term interval for the study was taken as symptoms arising more than four years after undergoing repairs. One of the reasons for this was patients who underwent a EVAR had an increased frequency of reinterventions, thereby narrowing the margin of long-term complications to open repairs [8].

Open repairs have greater mortality rates compared to EVAR. One of the reason for such increase in mortality is due to a higher prevalence of IC in open repair.. A retrospective study was done in 275 patients who have been treated for infrarenal aortic aneurysms, and morbidity and mortality were evaluated in those who developed IC. Out of 275 patients, 14 patients (5%) developed IC: four patients (1.8%) with EVAR and 10 patients (17.5%) with open repair [3]. Another study was done by Dr. Zhobin on a cohort of 3,486 patients, in which 11.6% underwent open repair and 88.4% were treated with EVAR. The incidence of acute IC was noted to be 2.2% with most cases seen in open repair (5.2%) and only a small number of cases in EVAR (1.8%). The overall prevalence rate of IC was estimated to be 2.9% [4]. Comparable results were reported in a study having a sample size of 9,145 patients who were operated for AAA. Of 9,145 patients, 8,248 were diagnosed with intact AAA (iAAA) and 897 with ruptured AAA (rAAA). 97 (1.2%) patients with iAAA presented with IC and 95 patients (10.6%) with rAAA came back positive for the same. When we classify these results based on repairs, among ruptured aneurysms, 12.8% developed IC post open surgical repair whereas only 3.7% presented with IC after EVAR [5]. A prospective study done through the Swedish vascular registry strongly claims preoperative shock and emergency surgery as being vital risk factors associated with IC [9].

 We will lay more emphasis on the study papers five and four because of their large patient population and attention to the details which pronounce them relevant to our literature review. IC may not be a prevalent complication, but what makes it dangerous is the high mortality associated with it. In patients with acute postoperative IC, the mortality rate reached an astonishing 41.2% in iAAA and 64.2% in rAAA. These values remained stable even after 90 days of diagnosis [5]. Dr. Zhobin’s retrospective study supports this incidence with an observed mortality rate of 38.7%. A comparatively lower rate was accounted for, likely due to the greater proportion of patients who underwent EVAR (88.4%) [4]. The length of hospital stay (LOS) was longer in patients with IC than in the subset without IC, with an estimated average of 12 more days in IAAA and five extra days in rAAA. These results were consistent with Dr. Zhobin’s findings with a mean difference of 12 days [4,5]. The Kaplan-Meier survival chart, upon analysis, shows worsening of the long-term survival after the occurrence of IC post-repair for both iAAA and rAAA [5].

One of the studies by Dr. Aday displays slight disparity, stating the researchers' inability to achieve statistical significance (p=0.393) between mortality rate in iAAA and rAAA. However, a positive association was observed in the percentage of mortality due to IC, which exceeded in rAAA (83.3%) when compared to unruptured AAA (62.5%) [3]. We believe that this discrepancy happened because of the study being limited to the small sample size of only 275 patients. We can infer from this data that IC may not be one of the most prevalent complications (2.9%), but it increases the rate of mortality and adds to the healthcare costs dramatically [4].

Now that we have discussed the mortality risk associated with IC and how this varies with different procedures and presentations, it is also inevitable to pay attention to the observational studies that identifies the risk factors which may play a role in the development of IC post repair.

When analyzing patients who developed IC in both open repair and EVAR, several risk factors were noted to be in common. Intraoperative and postoperative transfusion (p=0.01), ruptured aneurysm (p=0.01), and renal failure requiring dialysis (p=0.02) were significant indicators for IC. Being female and a presence of diabetes mellitus also showed a slight association with IC with a p-value of 0.04. When open repair and EVAR were observed individually, open repair, renal failure and ruptured aneurysm came out as predominant risk factors associated with IC. In EVAR, transfusions and ruptured aneurysm were found to be vital predictors for IC. This data has been summarized in Table 1 [4]. The Vascular Study group of New England database observed 8.9% of all rAAA and 0.5%-2.2% of all iAAA developing IC [5]. A separate study in 1998 by Elixhauser also supports the increased incidence of IC in female sex, advanced age group, and the volume of blood lost [10]. One of the reasons for ruptured aneurysm being a statistically significant predictor for both open repair and EVAR is due to the colon itself being susceptible to ischemia after rupture. A rupture facilitates hypoperfusion and altered mesenteric artery circulation, which are amplified by the preexisting atherosclerotic disease [11]. Table 1 provides a brief summary of the multivariate regression analysis performed by Dr. Zhobin on patients who developed IC after AAA repair between the year 2011 and 2012. The primary goal is to highlight the statistical significant risk factors which facilitated the progression of postoperative IC [4]. 

**Table 1:** Important predictors of ischemic colitis (IC) after aortic aneurysm repair Endovascular aneurysm repair (EVAR)

Risk factors associated with postoperative ischemic colitis (IC)	Open repair (p-value)	EVAR (p-value)
Renal failure	0.02	0.36
Bleeding disorder	0.03	0.6
Diabetes mellitus	0.01	0.31
Ruptured aneurysm	<0.01	0.04
Proximal extended aneurysm	0.02	0.22
Intraoperative/postoperative transfusion	0.23	<0.01
Emergency admission	0.58	0.04

A similar study in 1997 claimed perioperative shock as the most powerful risk factor. Renal disease was identified as an independent factor during multivariate analysis for IC [9]. Many studies have also shown consistency with the development of IC in the female gender and elderly population [5].

Patients who have undergone open repair displayed three times the likelihood of developing IC than patients with EVAR. However, after multivariate analysis, statistical significance was not identified between IC and open repair. One of the reasons for this is due to the presence of compounding variables which are naturally more prevalent in open repair. Open repair was directly correlated with an increased need for transfusion (intraoperative and preoperative), ruptured aneurysm, and proximal extension [4]. EVAR has extensively been opted for elective repairs. One of the benefits of EVAR is that it does not require aortic clamping and maintains the linear blood flow in the aorta. Prolonged cross-clamping and interference to the visceral flow increases the risk of IC [12]. Patients who underwent surgery for ruptured aneurysms were at a greater risk of substantial blood loss and hence in a state shock. More than 10 liters of blood loss increased the risk of ischemia hugely. One study indicates that around 30 % of rAAA required more than 10 units of blood and its components, whereas only 5% necessitated less than 10 units [9]. During the early stages of hypovolemic shock, visceral circulation is primarily affected which produces vasoconstriction and diminishes tissue perfusion causing ischemia [12]. Patients with chronic kidney disease requiring dialysis also exhibited correlation with IC. These patients are more prone to develop prolonged hemodynamic compromise especially during periods of shock (both perioperative and intraoperative), requiring vasopressors to maintain stability [9]. Retroperitoneal hematoma can produce hypoperfusion and increase abdominal compartment pressures due to intestinal traction, worsening the progression of IC [9,12].

Though, an increase in the occurrence of IC was noticed in a chronically occluded IMA or ligated IMA, a statistically significant association was not achieved with a p-value of 1 and 0.99, respectively [4]. Other risk factors that escalated the incidence of postoperative IC, though not statistically significant, were hypothermia (p=0.61), hypovolemia, and acidosis [12]. However, reimplantation of IMA and internal iliac embolization did not produce statistically significant data or increased the incidence of IC [4,12].

Despite IC being a cornerstone of research for the past 30 years, the incidence rates of this life-threatening complication have shown no signs of curtailment [5].

Due to vague and non-specific signs and symptoms, the diagnosis of IC can be delayed and thereby worsen the outcomes. This reiterates the importance of high index of suspicion especially in patient, presenting with risk factors as discussed. In a retrospective study done by Dr. Aday, abdominal pain (75% without rupture and 33.3% with rupture) and distention (50% with rupture and 66.7% without rupture) were the most common symptoms. Rectal bleeding was apparent in 12.5% of patients without rupture and 16.7% of patients with rupture [3]. A separate study reports bloody diarrhea as the most frequent finding. Cases with high suspicion were confirmed with colonoscopy [13]. A systematic review suggests close monitoring of patients as an essential tool particularly in emergencies (p < 0.001) and in patients with open repair [12]. EVAR should always be the primary choice for aneurysmal repair as it bypasses the complexities of open repair and decreases the possibility of IC.

It is essential that we reinforce the significance of the early predictors for IC and keep a high index of suspicion. Due to low prevalence rates of IC, the complication might be missed subconsciously and may not come up as a potential threat, even in the presence of risk factors [4]. Our goal is to bring more awareness to the clinicians and be mindful of the early signs and risk factors related to IC so that necessary intervention could be implemented in the early stages and curb the staggering mortality rates. There is a room for discussion about the effectiveness of EVAR and new advancements in minimally invasive procedures in reducing the rate of IC. Several studies have conflicting views and more data is needed to reach a definitive viewpoint.

## Conclusions

Our paper analyzed free articles including systematic reviews from several databases. Though most of the data we identified focused on risks factors and findings, necessary for early intervention, we failed to accumulate sufficient studies demonstrating statistical significance in the prevention of IC. Despite advancements in minimally invasive procedures, the data cannot provide a significant decline in the occurrence of IC rates in the predisposed groups.

It is inevitable to understand the merit of early identification of the disease. A high index of suspicion must be maintained amid relevant risk factors especially, ruptured aneurysm, intraoperative and perioperative shock, emergency repair, transfusion requirements, and renal failure. EVAR has shown evidence in reducing IC and must be considered for AAA repair if feasible. Early diagnosis allows time for better management plans and prolongs survival rates. Evidence demonstrates an increase in mortality after surgical laparotomy for IC. Poor tolerance of surgery in the elderly population along with underlying medical comorbidities leads to lengthy hospitalizations, medication overuse, poor recovery, and consequently increased death rates. The mortality rate associated with this complication is steep and clinicians must not wait for colonoscopy even if the patient presents with vague complaints like abdominal pain and distention, especially after emergencies and open repairs. We hope to raise and reinforce awareness towards this topic and improve longevity after aortic aneurysm repair through early identification and necessary intervention.
